# Supplementary material for: Konjac Glucomannan Counteracted the Side Effects of Excessive Exercise on Gut Microbiome, Endurance, and Strength in an Overtraining Mice Model
Source: Nutrients. 2023 Sep 29;15(19):4206. doi: 10.3390/nu15194206 (PMC10574454; doi:10.3390/nu15194206)
Supplement: Supplementary file 1 [file nutrients-15-04206-s001.zip › nutrients-2599053-supplementary.pdf]

Supplementary Data

Table S1. The chemical compounds used in this work with information from NCBI PubChem compound database and the supplier sources

Compound	CAS No.	CID	MW (g/mol)	InChIKey	IUPAC name	Source
Acetic acid	64-19-7	176	60.05	QTBSBXVTEAMEQO-UHFFFAOYSA-N	acetic acid	Aladdin® (Shanghai, China)
Propionic acid	79-09-4	1032	74.08	XBDQKXXYIPTUBI-UHFFFAOYSA-N	propanoic acid	Aladdin® (Shanghai, China)
n-Butyric acid	107-92-6	264	88.11	FERIUCNNQQJTOY-UHFFFAOYSA-N	butanoic acid	Aladdin® (Shanghai, China)
iso-Butyric acid	79-31-2	6590	88.11	KQNPFQTWMSNSAP-UHFFFAOYSA-N	2-methylpropanoic acid	Aladdin® (Shanghai, China)
n-valeric acid	109-52-4	7991	102.13	NQPDZGIKBAWPEJ-UHFFFAOYSA-N	pentanoic acid	Aladdin® (Shanghai, China)
iso-valeric acid	503-74-2	10430	102.13	GWYFCOCPABKNJV-	3-methylbutanoic acid	Aladdin® (Shanghai, China)

				UHFFFAOYSA-N		
n-hexanoic acid	142-62-1	8892	116.16	FUZZWVXGSFPDMH-UHFFFAOYSA-N	hexanoic acid	Aladdin® (Shanghai, China)
Iso-hexanoic acid	646-07-1	12587	116.16	FGKJLKRYENPLQH-UHFFFAOYSA-N	4-methylpentanoic acid	Aladdin® (Shanghai, China)
phosphoric acid	7664-38-2	1004	97.995	NBIIXXVUZAFLBC-UHFFFAOYSA-N	phosphoric acid	Aladdin® (Shanghai, China)
Ethyl acetate	141-78-6	8857	88.11	XEKOWRVHYACXOJ-UHFFFAOYSA-N	ethyl acetate	Merck® (Darmstadt, Germany)
Paraformaldehyde	50-00-0	712	30.026	WSFSSNUMVMOOMR-UHFFFAOYSA-N	formaldehyde	Macklin® (Shanghai, China)

CID: PubChem compound ID; InChIKey: International Chemical Identifier

Table S2. The formula of AIN93 purified diet used in the study

Ingredient	gram	kcal
Casein, 30Mesh	200	800
L-Cystine	3	12
Corn Starch	397	1590
Maltodextrin 10	132	528
Sucrose	100	400
Cellulose	50	0
Soybean Oil	70	630
t-Butylhydroquinone	0.014	0
Mineral Mix S10022M	35	0
Vitamin Mix V10037	10	40
Choline Bitartrate	2.5	0
Total	1000	3850
Macronutrients		
Protein	20.0 (gram%)	20.3 (Kcal%)
Carbohydrate	64.0 (gram%)	63.9 (Kcal%)
Fat	7.0 (gram%)	15.8 (Kcal%)
Total		100.0 (Kcal%)
kcal/gm	3.9	

Table S3. Histological scoring system

Histological score	Degree of inflammation	Infiltration of inflammatory cells	Degree of damage to the crypt	Crypt abscesses	Degree of submucosal edema	Reduction of goblet cells	Degree of epithelial hyperplasia
0	Normal	Normal	Normal	Normal	Normal	Normal	Normal
1	Mucosa	Unifocal	Basal 1/3 of crypt	Unifocal	Unifocal	Unifocal	Unifocal
2	Submucosa	Multifocal	Basal 2/3 of crypt	Multifocal	Multifocal	Multifocal	Multifocal
3	Muscular	Suffuse	Entire crypt		Suffuse	Suffuse	Suffuse
4	Serous		Damage to the crypt and ulceration				

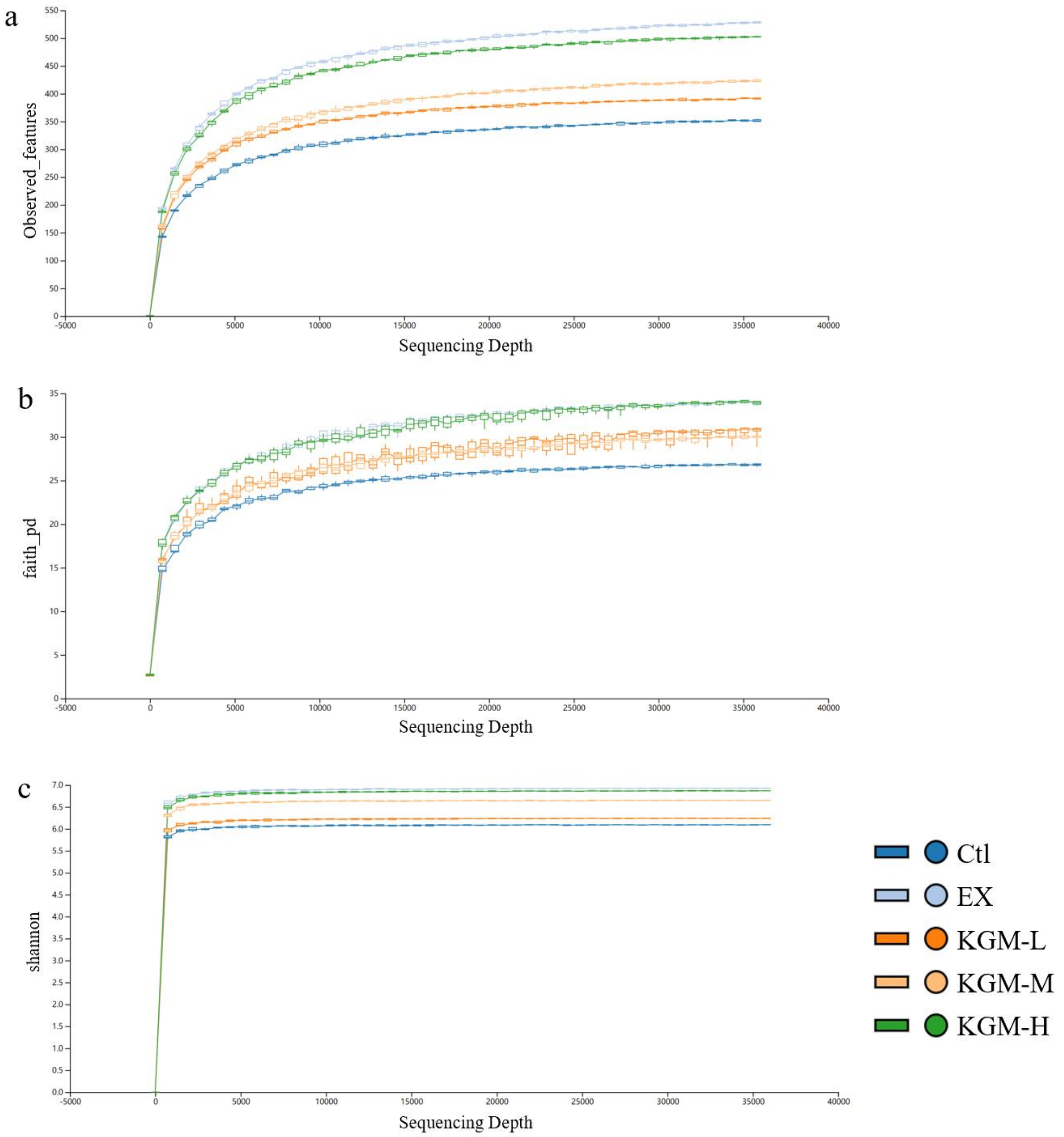

Figure S1. Rarefaction curves of alpha diversity on day 0. Ctl: control; EX: excessive exercise; KGM-L: low dose of KGM (1.25 g/L in drinking water) with excessive exercise; KGM-M: moderate dose (2.50 g/L in drinking water) of KGM with excessive exercise; KGM-H: high dose (5.00 g/L in drinking water) of KGM with excessive exercise.

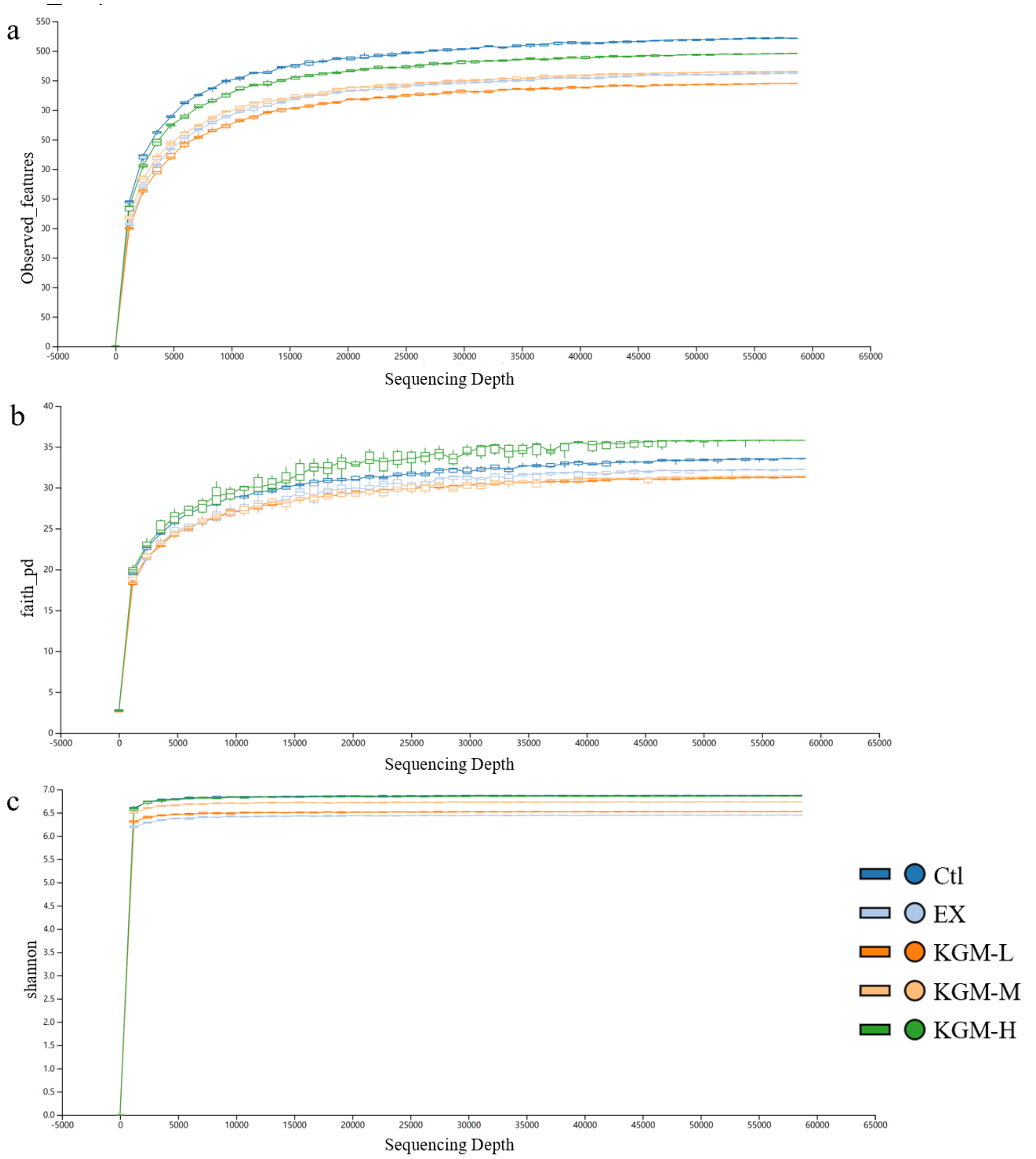

Figure S2. Rarefaction curves of alpha diversity on day 21. Ctl: control; EX: excessive exercise; KGM-L: low dose of KGM (1.25 g/L in drinking water) with excessive exercise; KGM-M: moderate dose (2.50 g/L in drinking water) of KGM with excessive exercise; KGM-H: high dose (5.00 g/L in drinking water) of KGM with excessive exercise.

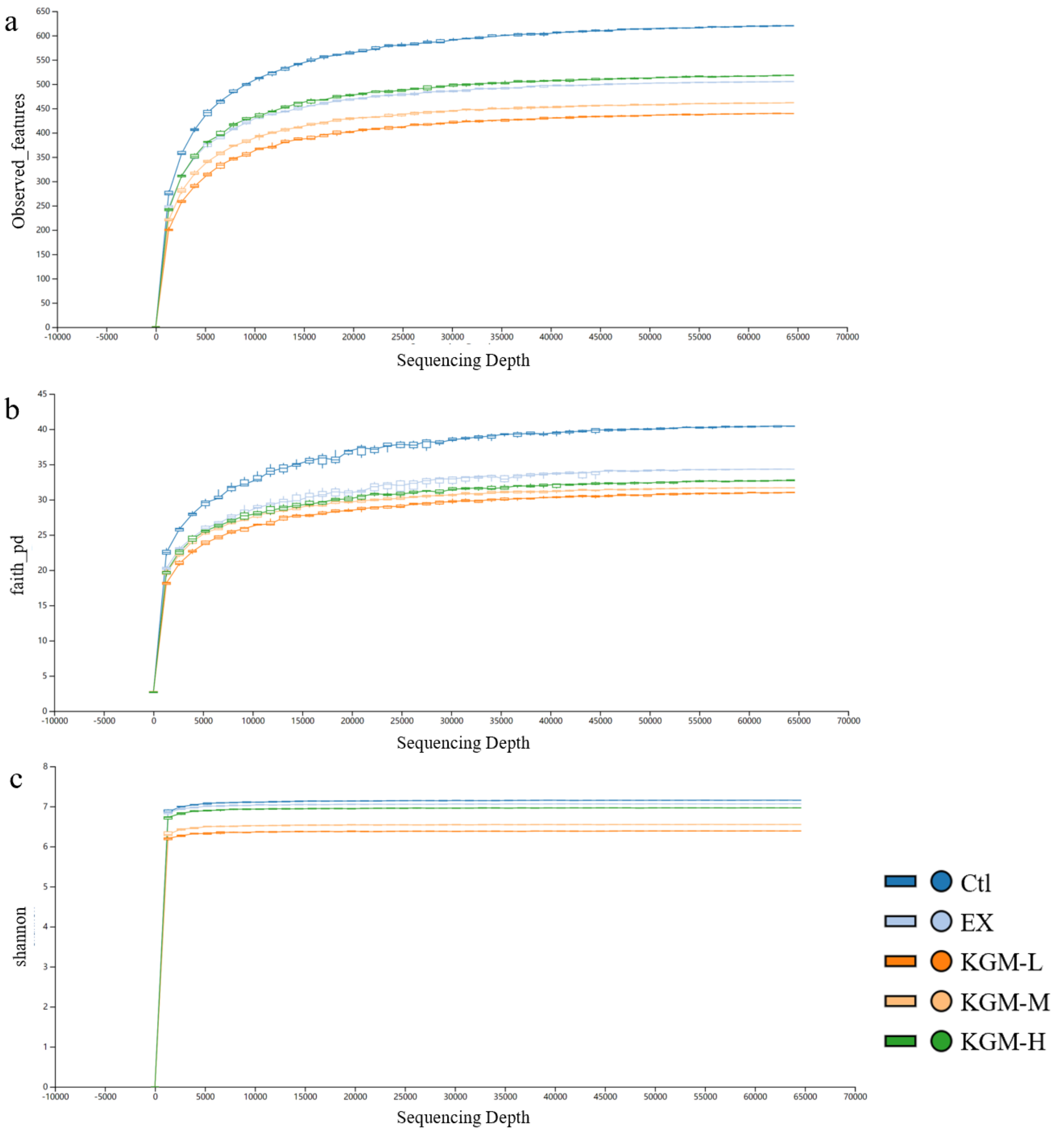

Figure S3. Rarefaction curves of alpha diversity on day 42. Ctl: control; EX: excessive exercise; KGM-L: low dose of KGM (1.25 g/L in drinking water) with excessive exercise; KGM-M: moderate dose (2.50 g/L in drinking water) of KGM with excessive exercise; KGM-H: high dose (5.00 g/L in drinking water) of KGM with excessive exercise.

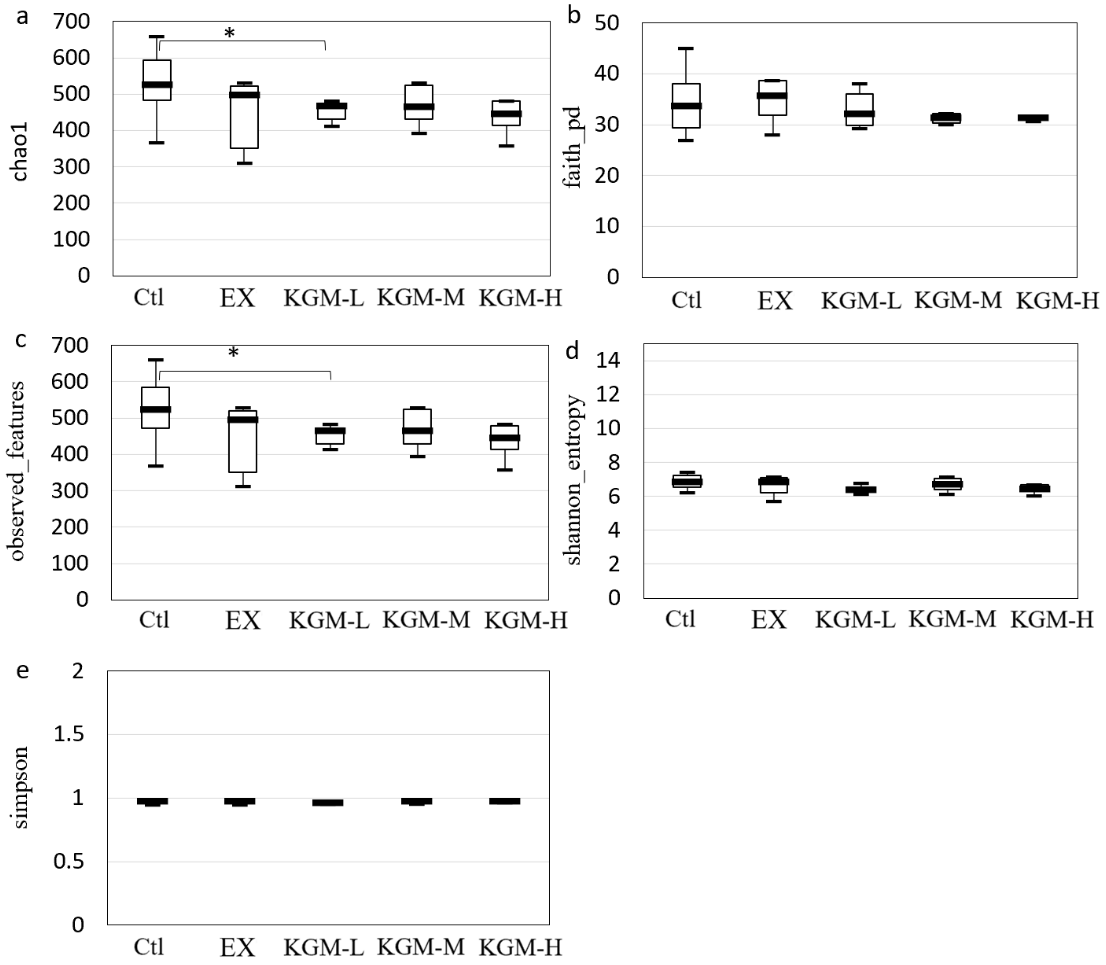

Figure S4. The alpha-diversity indices of fecal microbiome on day 21. The star indicates the significant difference between two groups. Asterisks indicate significant difference between two groups, Krustal-Wallis test, $p < 0.05$, N = 6. Ctl: control; EX: excessive exercise; KGM-L: low dose of KGM (1.25 g/L in drinking water) with excessive exercise; KGM-M: moderate dose (2.50 g/L in drinking water) of KGM with excessive exercise; KGM-H: high dose (5.00 g/L in drinking water) of KGM with excessive exercise.

Table S4. The relative abundance of top 20 taxa in feces at different levels on day 21. Ctl: control; EX: excessive exercise; KGM-L: low dose of KGM (1.25 g/L in drinking water) with excessive exercise; KGM-M: moderate dose (2.50 g/L in drinking water) of KGM with excessive exercise; KGM-H: high dose (5.00 g/L in drinking water) of KGM with excessive exercise.

	**Ctl**	**EX**	**KGM-L**	**KGM-M**	**KGM-H**
**Phylum level**					
Bacteroidetes	53.70 ± 7.93 b	58.29 ± 10.51 ab	66.00 ± 9.66 a	66.46 ± 7.38 a	68.58 ± 8.47 a
Firmicutes	31.63 ± 6.94 a	29.66 ± 7.24 a	23.60 ± 8.25 a	24.12 ± 7.92 a	22.62 ± 6.07 b
Proteobacteria	12.24 ± 7.41	8.77 ± 3.59	7.84 ± 3.39	7.38 ± 2.10	7.28 ± 5.21
Actinobacteria	1.74 ± 1.26	1.04 ± 0.58	1.86 ± 1.99	0.85 ± 0.60	0.69 ± 0.47
Tenericutes	0.57 ± 0.19 b	1.74 ± 1.12 a	0.48 ± 0.19 b	0.99 ± 0.85 ab	0.62 ± 0.24 b
Deferribacteres	0.06 ± 0.03 a	0.34 ± 0.29 ab	0.01 ± 0.02 b	0.10 ± 0.11 ab	0.05 ± 0.08 ab
TM7	0.02 ± 0.02	0.08 ± 0.12	0.04 ± 0.05	0.08 ± 0.08	0.08 ± 0.05
Cyanobacteria	0.01 ± 0.01	0.03 ± 0.03	0.16 ± 0.28	0.03 ± 0.02	0.04 ± 0.03
unclassified	0.04 ± 0.05	0.03 ± 0.02	0.01 ± 0.01	0.01 ± 0.01	0.01 ± 0.01
Verrucomicrobia	0.00 ± 0.01	0.01 ± 0.02	0.01 ± 0.01	0.00 ± 0.00	0.00 ± 0.01
Chlamydiae	0.00 ± 0.00	0.00 ± 0.00	0.00 ± 0.01	0.00 ± 0.00	0.01 ± 0.01
OD1	0.00 ± 0.00	0.01 ± 0.01	0.00 ± 0.00	0.00 ± 0.00	0.00 ± 0.00
Acidobacteria	0.00 ± 0.00	0.00 ± 0.01	0.00 ± 0.00	0.00 ± 0.00	0.01 ± 0.01
Chloroflexi	0.00 ± 0.00	0.00 ± 0.00	0.00 ± 0.00	0.00 ± 0.00	0.00 ± 0.01
Fusobacteria	0.00 ± 0.00	0.00 ± 0.00	0.00 ± 0.00	0.00 ± 0.00	0.00 ± 0.00
Gemmatimonadetes	0.00 ± 0.00	0.00 ± 0.00	0.00 ± 0.00	0.00 ± 0.00	0.00 ± 0.00
**Genus level**					
unclassified	53.80 ± 5.28	51.19 ± 4.90	49.38 ± 6.22	51.27 ± 10.07	53.08 ± 6.55
Bacteroides	7.28 ± 1.87 b	8.71 ± 3.91 b	14.41 ± 4.86 a	12.12 ± 6.32 ab	11.58 ± 3.27 ab
Prevotella	4.58 ± 3.00 b	10.21 ± 7.72 a	5.49 ± 2.12 ab	4.93 ± 1.09 b	7.53 ± 3.23 ab

Prevotellaceae _Prevotella	2.62 ± 2.72 b	2.69 ± 1.71 b	5.83 ± 2.58 ab	6.72 ± 3.25 a	8.21 ± 5.03 a
Flexispira	4.21 ± 2.41	4.73 ± 2.86	5.82 ± 2.85	4.78 ± 2.41	5.53 ± 5.10
Allobaculum	3.43 ± 1.99	7.58 ± 4.49	5.33 ± 4.03	4.17 ± 2.15	3.51 ± 2.12
Lactobacillus	8.53 ± 4.28 a	2.12 ± 1.82 b	3.37 ± 2.38 b	4.00 ± 4.34 b	2.21 ± 0.69 b
Parabacteroides	1.42 ± 0.46 bc	1.33 ± 0.60 c	2.69 ± 0.69 a	2.63 ± 0.99 a	2.16 ± 0.58 ab
Oscillospira	1.79 ± 0.46	2.42 ± 1.61	0.93 ± 0.44	1.79 ± 0.86	1.04 ± 0.22
Pseudomonas	4.98 ± 7.76	0.94 ± 0.74	0.35 ± 0.24	0.69 ± 0.74	0.42 ± 0.35
Sutterella	1.51 ± 0.89 ab	2.01 ± 0.65 a	1.34 ± 0.74 ab	1.33 ± 0.50 ab	0.93 ± 0.27 b
Coprococcus	1.40 ± 0.75	2.00 ± 1.79	0.55 ± 0.29	0.73 ± 0.38	0.45 ± 0.19
Ruminococcaceae _Ruminococcus	0.63 ± 0.35	0.72 ± 0.43	0.52 ± 0.28	0.52 ± 0.26	0.46 ± 0.13
Rikenella	0.20 ± 0.13 b	0.32 ± 0.15 b	0.53 ± 0.19 a	0.70 ± 0.30 a	0.41 ± 0.20 b
Odoribacter	0.18 ± 0.04 b	0.41 ± 0.21 a	0.32 ± 0.10 ab	0.58 ± 0.32 a	0.38 ± 0.11 a
Bifidobacterium	0.16 ± 0.17 b	0.11 ± 0.06 b	1.31 ± 1.70 a	0.16 ± 0.16 b	0.09 ± 0.06 b
Erysipelotrichaceae _Clostridium	0.25 ± 0.07	0.26 ± 0.12	0.40 ± 0.20	0.54 ± 0.77	0.29 ± 0.19
Paraprevotella	0.26 ± 0.09	0.22 ± 0.17	0.26 ± 0.09	0.45 ± 0.35	0.33 ± 0.19
Adlercreutzia	0.24 ± 0.18	0.32 ± 0.32	0.22 ± 0.13	0.43 ± 0.31	0.30 ± 0.24
AF12	0.17 ± 0.02 b	0.27 ± 0.08 a	0.19 ± 0.03 b	0.24 ± 0.04 ab	0.18 ± 0.06 b
Other	2.38 ± 2.46	1.46 ± 0.93	0.77 ± 0.18	1.23 ± 0.51	0.91 ± 0.35

Data are shown in average ± standard deviation. Different letters indicate the significant difference among different groups for the same index, ANOVA with LSD or Tamhane T2 post-hoc test, $p < 0.05$. N = 6.

Table S5. The relative abundance of top 20 taxa in feces at different levels on day 42. Ctl: control; EX: excessive exercise; KGM-L: low dose of KGM (1.25 g/L in drinking water) with excessive exercise; KGM-M: moderate dose (2.50 g/L in drinking water) of KGM with excessive exercise; KGM-H: high dose (5.00 g/L in drinking water) of KGM with excessive exercise.

	**Ctl**	**EX**	**KGM-L**	**KGM-M**	**KGM-H**
**Phylum**					
Bacteroidetes	36.92 ± 6.16 b	61.34 ± 7.15 a	48.06 ± 14.59 ab	60.14 ± 7.03 a	62.07 ± 7.54 a
Firmicutes	49.91 ± 3.89 a	29.57 ± 5.82 b	37.08 ± 12.69 b	33.16 ± 6.39 b	28.94 ± 5.88 b
Proteobacteria	7.47 ± 4.42 ab	5.71 ± 1.10 ab	9.33 ± 5.87 a	4.29 ± 1.59 b	5.74 ± 2.27 ab
Actinobacteria	3.79 ± 3.91 ab	2.64 ± 1.43 ab	4.26 ± 1.78 a	1.34 ± 0.53 b	2.25 ± 1.24 ab
Tenericutes	1.28 ± 0.60	0.37 ± 0.13	1.05 ± 1.15	0.88 ± 0.82	0.76 ± 0.31
TM7	0.35 ± 0.27	0.27 ± 0.27	0.08 ± 0.11	0.06 ± 0.06	0.14 ± 0.04
Deferribacteres	0.19 ± 0.17	0.05 ± 0.03	0.02 ± 0.02	0.04 ± 0.05	0.04 ± 0.03
Chlamydiae	0.02 ± 0.02	0.03 ± 0.02	0.10 ± 0.17	0.04 ± 0.02	0.03 ± 0.02
Cyanobacteria	0.01 ± 0.01 ab	0.00 ± 0.00 b	0.01 ± 0.00 a	0.02 ± 0.03 ab	0.02 ± 0.01 ab
unclassified	0.02 ± 0.01 a	0.01 ± 0.01 bc	0.00 ± 0.00 c	0.01 ± 0.01 ab	0.01 ± 0.01 bc
Verrucomicrobia	0.02 ± 0.04	0.00 ± 0.01	0.00 ± 0.00	0.00 ± 0.00	0.00 ± 0.00
Acidobacteria	0.01 ± 0.01	0.00 ± 0.00	0.00 ± 0.00	0.01 ± 0.03	0.00 ± 0.00
Chloroflexi	0.01 ± 001	0.00 ± 0.00	0.00 ± 0.00	0.00 ± 0.01	0.00 ± 0.00
OD1	0.00 ± 0.00	0.00 ± 0.00	0.00 ± 0.00	0.00 ± 0.00	0.00 ± 0.00
Armatimonadetes	0.00 ± 0.00	0.00 ± 0.00	0.00 ± 0.00	0.00 ± 0.00	0.00 ± 0.00
**Family**					
S24_7	26.21 ± 3.36 c	40.58 ± 3.76 a	30.64 ± 9.47 bc	37.06 ± 5.62 ab	42.48 ± 5.22 a
Erysipelotrichaceae	18.54 ± 8.74	9.80 ± 3.55	23.67 ± 12.80	9.03 ± 3.90	11.35 ± 3.87
unclassified	12.02 ± 1.98 a	8.13 ± 2.29 b	4.44 ± 1.97 b	4.80 ± 1.09 b	6.83 ± 1.88 b
Bacteroidaceae	2.22 ± 0.77 b	5.97 ± 2.50 a	8.24 ± 4.71 ab	10.41 ± 2.88 a	7.13 ± 0.66 a

Paraprevotellaceae	5.12 ± 2.65 ab	8.69 ± 5.05 a	3.98 ± 1.65 b	7.08 ± 3.68 ab	7.96 ± 1.69 ab
Lachnospiraceae	11.21 ± 5.20 a	6.74 ± 3.00 b	2.96 ± 1.35 c	3.18 ± 2.13 bc	4.88 ± 1.64 bc
Lactobacillaceae	1.17 ± 1.09 b	2.03 ± 2.36 b	3.97 ± 3.27 b	13.79 ± 9.69 a	2.69 ± 1.80 b
Ruminococcaceae	7.14 ± 2.02 a	3.02 ± 0.83 b	2.09 ± 0.96 b	2.37 ± 0.84 b	3.40 ± 1.03 b
Helicobacteraceae	4.77 ± 4.18	1.87 ± 1.47	3.59 ± 2.65	1.36 ± 0.44	2.89 ± 2.48
Alcaligenaceae	0.68 ± 0.23 b	3.01 ± 0.62 a	4.55 ± 2.75 ab	2.04 ± 1.33 ab	1.87 ± 1.07 ab
Coriobacteriaceae	3.11 ± 2.89	1.37 ± 0.73	3.04 ± 1.49	1.25 ± 0.48	2.03 ± 1.30
Prevotellaceae	1.01 ± 0.49 b	3.51 ± 3.46 a	2.44 ± 2.14 ab	2.06 ± 2.10 ab	1.24 ± 0.64 ab
Porphyromonadaceae	0.67 ± 0.21 b	1.29 ± 0.37 ab	1.84 ± 0.84 ab	1.76 ± 0.73 ab	1.67 ± 0.38 a
Rikenellaceae	1.04 ± 0.20 a	0.88 ± 0.22 ab	0.54 ± 0.11 b	1.02 ± 0.40 ab	0.94 ± 0.27 ab
Desulfovibrionaceae	1.08 ± 0.41 a	0.61 ± 0.28 ab	0.50 ± 0.39 b	0.60 ± 0.50 ab	0.77 ± 0.39 ab
Bifidobacteriaceae	0.53 ± 0.76 ab	1.26 ± 0.89 ab	1.22 ± 1.25 ac	0.07 ± 0.07 b	0.22 ± 0.07 b
Mycoplasmataceae	0.87 ± 0.67	0.14 ± 0.06	0.90 ± 1.22	0.68 ± 0.79	0.52 ± 0.29
Odoribacteraceae	0.23 ± 0.06	0.28 ± 0.05	0.22 ± 0.04	0.56 ± 0.38	0.46 ± 0.14
F16	0.35 ± 0.27	0.27 ± 0.27	0.08 ± 0.11	0.06 ± 0.06	0.14 ± 0.04
Pseudomonadaceae	0.35 ± 0.21	0.15 ± 0.11	0.09 ± 0.03	0.17 ± 0.13	0.11 ± 0.05
Other	1.67 ± 1.49	0.39 ± 0.10	1.01 ± 1.38	0.66 ± 0.59	0.41 ± 0.10
**Genus**					
unclassified	59.56 ± 6.53 ab	58.68 ± 7.26 ab	43.57 ± 11.57 ab	47.87 ± 5.45 b	58.80 ± 4.25 a
Allobaculum	12.27 ± 6.94 b	7.16 ± 2.43 b	22.08 ± 13.08 a	7.62 ± 3.66 b	9.02 ± 3.41 b
Bacteroides	2.22 ± 0.77 b	5.97 ± 2.50 ab	8.24 ± 4.71 ab	10.41 ± 2.88 a	7.13 ± 0.66 a
Prevotella	4.95 ± 2.69 ab	8.46 ± 4.96 a	3.78 ± 1.61 b	6.81 ± 3.68 ab	7.73 ± 1.63 a
Lactobacillus	1.17 ± 1.09 b	2.03 ± 2.36 b	3.97 ± 3.27 b	13.79 ± 9.68 a	2.69 ± 1.80 b
Flexispira	4.76 ± 4.18	1.87 ± 1.47	3.59 ± 2.65	1.36 ± 0.45	2.88 ± 2.48
Sutterella	0.67 ± 0.23 b	3.01 ± 0.62 a	4.55 ± 2.75 ab	2.04 ± 1.33 ab	1.87 ± 1.07 ab
Prevotellaceae_	1.01 ± 0.49	3.51 ± 3.46	2.44 ± 2.14	2.06 ± 2.10	1.24 ± 0.64

Prevotella					
Oscillospira	3.41 ± 1.32	1.51 ± 0.57	1.07 ± 0.53	1.08 ± 0.41	1.49 ± 0.34
Parabacteroides	0.67 ± 0.21 b	1.29 ± 0.37 ab	1.84 ± 0.84 ab	1.76 ± 0.73 ab	1.67 ± 0.38 a
Ruminococcaceae _Ruminococcus	1.95 ± 0.77 a	0.98 ± 0.24 bc	0.60 ± 0.35 c	0.73 ± 0.26 bc	1.17 ± 0.42 b
Adlercreutzia	1.74 ± 2.00	0.60 ± 0.34	0.62 ± 0.35	0.48 ± 0.26	0.65 ± 0.42
Coprococcus	1.23 ± 0.59 a	1.11 ± 0.44 a	0.33 ± 0.15 b	0.69 ± 0.69 ab	0.70 ± 0.20 ab
Bifidobacterium	0.53 ± 0.76 ab	1.26 ± 0.89 a	0.66 ± 0.70 ab	0.07 ± 0.07 b	0.22 ± 0.07 b
Rikenella	0.33 ± 0.14	0.41 ± 0.20	0.26 ± 0.03	0.48 ± 0.29	0.40 ± 0.17
Odoribacter	0.23 ± 0.06	0.28 ± 0.05	0.22 ± 0.04	0.56 ± 0.38	0.46 ± 0.14
Erysipelotrichaceae _Clostridium	0.27 ± 0.15	0.43 ± 0.26	0.25 ± 0.11	0.41 ± 0.22	0.32 ± 0.14
Desulfovibrio	0.44 ± 0.12	0.26 ± 0.20	0.26 ± 0.12	0.29 ± 0.17	0.43 ± 0.35
Paraprevotella	0.18 ± 0.05	0.23 ± 0.14	0.21 ± 0.12	0.27 ± 0.06	0.23 ± 0.08
AF12	0.28 ± 0.10 ab	0.23 ± 0.07 a	0.10 ± 0.03 b	0.19 ± 0.03 a	0.21 ± 0.04 a
Other	2.13 ± 1.40 a	0.75 ± 0.21 b	1.36 ± 1.78 ab	1.06 ± 0.62 ab	0.70 ± 0.19 ab

Data are shown in average ± standard deviation. Different letters indicate the significant difference among different groups for the same index, ANOVA with LSD or Tamhane T2 post-hoc test, $p < 0.05$. N = 6.

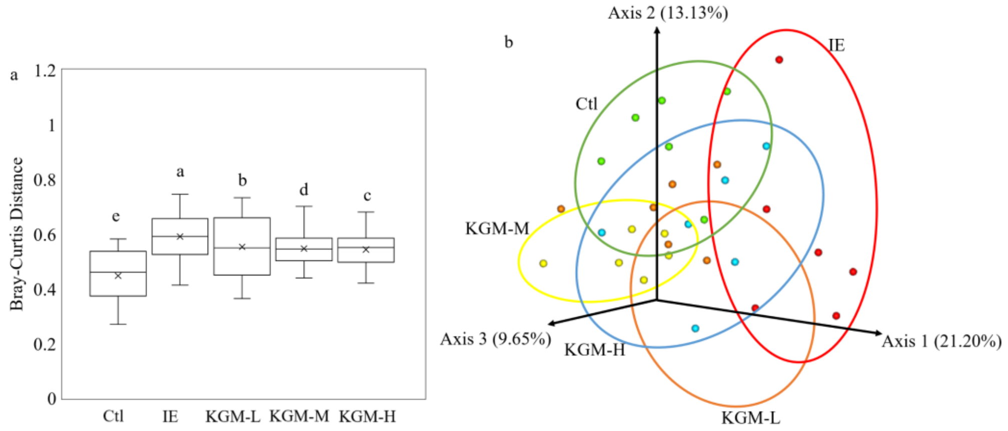

Figure S5. (a) The Bray-Curtis distances between the Ctl group and five groups, (b) the principal component analysis (PCoA) on Day 0. Different letters indicate significant difference between two groups, permutational multivariate analysis of variance test, $p < 0.05$, N = 6. Ctl: control; EX: excessive exercise; KGM-L: low dose of KGM (1.25 g/L in drinking water) with excessive exercise; KGM-M: moderate dose (2.50 g/L in drinking water) of KGM with excessive exercise; KGM-H: high dose (5.00 g/L in drinking water) of KGM with excessive exercise.

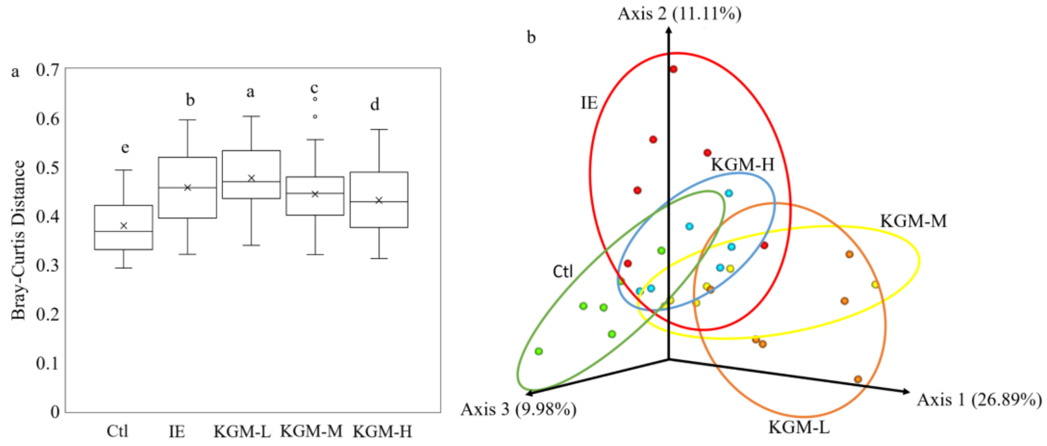

Figure S6. (a)The Bray-Curtis distances between the Ctl group and five groups, (b) the principal component analysis (PCoA) on Day 21. Different letters indicate significant difference between two groups, permutational multivariate analysis of variance test, $p < 0.05$, N = 6. Ctl: control; EX: excessive exercise; KGM-L: low dose of KGM (1.25 g/L in drinking water) with excessive exercise; KGM-M: moderate dose (2.50 g/L in drinking water) of KGM with excessive exercise; KGM-H: high dose (5.00 g/L in drinking water) of KGM with excessive exercise.

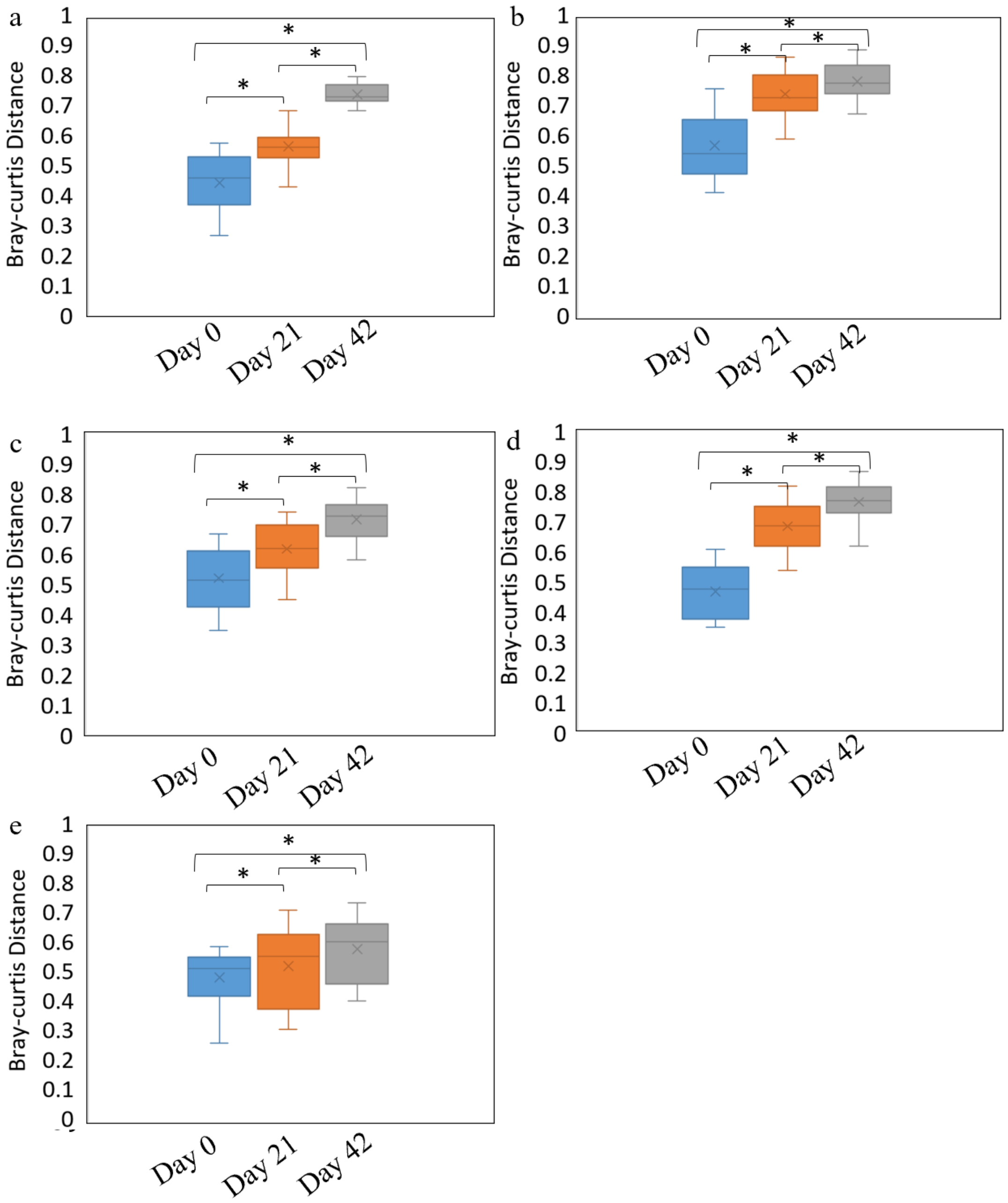

Figure S7. Bray-curtis distance from day 0, 21 and 42 to Day 0. (a) Ctl: control, (b) EX: excessive exercise, (c) KGM-L: low dose of KGM (1.25 g/L in drinking water) with excessive exercise, (d) KGM-M: moderate dose (2.50 g/L in drinking water) of KGM with excessive exercise, and (e) KGM-H: high dose (5.00 g/L in drinking water) of KGM with excessive exercise. Asterisks indicate significant difference, $p < 0.05$, analysis of similarities (PERMANOVA).
